# Youth and Age (Poetry)

**Published:** 1881-07

**Authors:** 


### YOUTH AND AGE.

[The following selections from Longfellow's "Morituri Salutamus" are beautiful descriptions of youth and age, with all their differences.]

How beautiful is youth! how bright it gleams,
With its illusions, aspirations, dreams!
Book of Beginnings, Story without End,
Each maid a heroine, each man a friend!
Aladdin's Lamp, and Fortunatus' purse
That holds the treasures of the universe!
All possibilities are in its hands,
No danger daunts it, and no foe withstands;
In its sublime audacity of faith,
"Be thou removed!" it to the mountain saith,
And with ambitious feet, secure and proud,
Ascends the ladder leaning on the cloud!

---

As the barometer foretells the storm
While still the skies are clear, the weather warm,
So something in us, as old age draws near,
Betrays the pressure of the atmosphere,
The nimble mercury, ere we are aware,
Descends the elastic ladder of the air;
The tell-tale blood in artery and vein
Sinks from the higher levels in the brain;
Whatever poet, orator or sage
May say of it, old age is still old age.
It is the waning, not the crescent moon,
The dusk of evening, not the blaze of noon;
It is not strength, but weakness; not desire,
But its surcease; not the fierce heat of fire,
The burning and consuming element,
But that of ashes and of embers spent,
In which some living sparks we still discern;
Enough to warm, but not enough to burn.
What then? Shall we sit idly down and say
The night has come, it is no longer day?
The night hath not yet come; we are not quite
Cut off from labor by the falling light;
Something remains for us to do or dare;
Even the oldest trees some fruit may bear;
For age is opportunity no less
Than youth itself, though in another dress;
And as the evening twilight fades away
The sky is filled with stars, invisible by day.

---

"DO BEES THINK?" is the conundrum that is bothering the pates of the etymologists. The action of bees is so sudden that it is impossible to believe that they think. If they considered—but never mind.—*New Haven Register.*

